# Addition of zoledronic acid to neoadjuvant chemotherapy is not beneficial in patients with HER2-negative stage II/III breast cancer: 5-year survival analysis of the NEOZOTAC trial (BOOG 2010-01)

**DOI:** 10.1186/s13058-019-1180-6

**Published:** 2019-08-28

**Authors:** Stefanie de Groot, Hanno Pijl, Ayoub Charehbili, Saskia van de Ven, Vincent T. H. B. M. Smit, Elma Meershoek-Klein Kranenbarg, Joan B. Heijns, Laurence J. C. van Warmerdam, Lonneke W. Kessels, M. Wouter Dercksen, Manon J. A. E. Pepels, Hanneke W. M. van Laarhoven, Birgit E. P. J. Vriens, Hein Putter, Marta Fiocco, Gerrit-Jan Liefers, Jacobus J. M. van der Hoeven, Johan W. R. Nortier, Judith R. Kroep, on behalf of the Dutch Breast Cancer Research Group

**Affiliations:** 1 Department of Medical Oncology, Leiden University Medical Center, Albinusdreef 2, Leiden, P.O. Box 9600, 2300 RC Leiden, The Netherlands; 2 Department of Endocrinology, Leiden University Medical Center, Leiden, The Netherlands; 3 Department of Radiotherapy, University Medical Center Utrecht, Utrecht, The Netherlands; 4 Department of Pathology, Leiden University Medical Center, Leiden, The Netherlands; 5 Department of Surgery, Leiden University Medical Center, Leiden, The Netherlands; 6 Department of Medical Oncology, Amphia hospital, Breda, The Netherlands; 7 Department of Clinical Oncology, Catharina Ziekenhuis, Eindhoven, The Netherlands; 8 Department of Medical Oncology, Deventer hospital, Deventer, The Netherlands; 9 Department of Clinical Oncology, Maxima Medisch Centrum, Veldhoven, The Netherlands; 10 Department of Clinical Oncology, Elkerliek Ziekenhuis, Helmond, The Netherlands; 11 Department of Medical Oncology, Cancer Center Amsterdam, Amsterdam Medical Centers, University of Amsterdam, Amsterdam, The Netherlands; 12 Department of Medical Statistics and Bioinformatics, Leiden University Medical Center, Leiden, The Netherlands; 13 Mathematical Department, Leiden University, Leiden, The Netherlands; 14 Department of Clinical Oncology, Radboud University, Nijmegen, The Netherlands; 15 Dutch Breast Cancer Research Group (BOOG), Amsterdam, The Netherlands

**Keywords:** Breast cancer, Neoadjuvant chemotherapy, Zoledronic acid, Survival, Insulin, IGF-1R

## Abstract

**Background:**

Adjuvant bisphosphonates are associated with improved breast cancer survival in postmenopausal patients. Addition of zoledronic acid (ZA) to neoadjuvant chemotherapy did not improve pathological complete response in the phase III NEOZOTAC trial. Here we report the results of the secondary endpoints, disease-free survival, (DFS) and overall survival (OS).

**Patients and methods:**

Patients with HER2-negative, stage II/III breast cancer were randomized to receive the standard 6 cycles of neoadjuvant TAC (docetaxel/doxorubicin/cyclophosphamide) chemotherapy with or without 4 mg intravenous (IV) ZA administered within 24 h of chemotherapy. This was repeated every 21 days for 6 cycles. Cox regression models were used to evaluate the effect of ZA and covariates on DFS and OS. Regression models were used to examine the association between insulin, glucose, insulin growth factor-1 (IGF-1) levels, and IGF-1 receptor (IGF-1R) expression with survival outcomes.

**Results:**

Two hundred forty-six women were eligible for inclusion. After a median follow-up of 6.4 years, OS for all patients was significantly worse for those who received ZA (HR 0.468, 95% CI 0.226–0.967, *P* = 0.040). DFS was not significantly different between the treatment arms (HR 0.656, 95% CI 0.371–1.160, *P* = 0.147). In a subgroup analysis of postmenopausal women, no significant difference in DFS or OS was found for those who received ZA compared with the control group (HR 0.464, 95% CI 0.176–1.222, *P* = 0.120; HR 0.539, 95% CI 0.228–1.273, *P* = 0.159, respectively). The subgroup analysis of premenopausal patients was not significantly different for DFS and OS ((HR 0.798, 95% CI 0.369–1.725, *P* = 0.565; HR 0.456, 95% CI 0.156–1.336, *P* = 0.152, respectively). Baseline IGF-1R expression was not significantly associated with DFS or OS. In a predefined additional study, lower serum levels of insulin were associated with improved DFS (HR 1.025, 95% CI 1.005–1.045, *P =* 0.014).

**Conclusions:**

Our results suggest that ZA in combination with neoadjuvant chemotherapy was associated with a worse OS in breast cancer (both pre- and postmenopausal patients). However, in a subgroup analysis of postmenopausal patients, ZA treatment was not associated with DFS or OS. Also, DFS was not significantly different between both groups. IGF-1R expression in tumor tissue before and after neoadjuvant treatment did not predict survival.

**Trial registration:**

ClinicalTrials.gov, NCT01099436, April 2010.

## Introduction

Bisphosphonates (BPs) act to suppress bone resorption by inducing osteoclast apoptosis [1, 2]. BPs are indicated for treatment and prevention of osteoporosis and prevention of skeletal-related events due to metastasis of solid tumors or multiple myeloma [3]. Results of the meta-analysis of the Early Breast Cancer Trialists’ Collaborative Group (EBCTCG) showed that adjuvant BPs were associated with decreased fracture rate, as well as improved breast cancer survival and bone metastasis risk. These benefits were only found in postmenopausal (natural or induced) women in a subgroup analysis [4]. The benefits may be explained by the increased bone resorption in postmenopausal patients, as BP prevented tumor growth in bone in a postmenopausal model but not in a premenopausal model [5]. Currently, BPs are considered as a part of the adjuvant breast cancer treatment in postmenopausal patients and patients receiving ovarian suppression therapy [6]. The exact mechanism of the anti-tumor effect of BPs is unknown. However, the following mechanisms have been proposed [7], BPs may (1) prevent tumors cells from metastasizing to the bone by decreasing bone turnover [8], (2) change the bone micro-environment by reducing growth factors such as insulin-like growth factor-1 (IGF-1) and insulin and thereby inhibit proliferation [9–12], (3) have immunomodulatory properties by activating γδ T cells [13, 14] and recruiting tumor-associated macrophages [15, 16], (4) reduce angiogenic factors [17, 18], and/or (5) kill dormant disseminated tumor cells [19, 20]. BP was reported to improve the tumor response when combined with doxorubicin in an experimental breast cancer model [21]. Moreover, adding a BP to neoadjuvant chemotherapy in breast cancer patients resulted in a significantly lower residual invasive tumor size and a non-significantly higher pathological complete response (pCR) rate in an exploratory evaluation of the AZURE trial [22].

Clinically, in our phase III randomized NEOZOTAC study examining the effect of zolendronic acid (ZA) in addition to neoadjuvant TAC chemotherapy in HER2 negative early breast cancer, ZA did not improve the primary endpoint, pathological complete response (pCR) [23]. A subsequent meta-analysis did not show a significant increase in pCR rate when adding a BP to neoadjuvant chemotherapy in patients with early breast cancer [16, 24]. In this paper, we report the secondary endpoints of disease-free survival (DFS) and overall survival (OS) from the NEOZOTAC study [23].

Additionally, we report associations between the IGF-1 receptor (IGF-1R) expression and the concentrations of circulating growth factors such as insulin and IGF-1, and survival. IGF-1R and insulin receptor isoform A (IR-A) are frequently upregulated in breast cancer [25, 26]. Both receptors activate the Ras/mitogen-activated protein kinase (MAPK) and phosphatidylinositol-3-kinase (PI3K)/AKT pathways, through which cell proliferation is stimulated and apoptosis is inhibited [27].

## Methods

### Study population

**Fig. 1:**
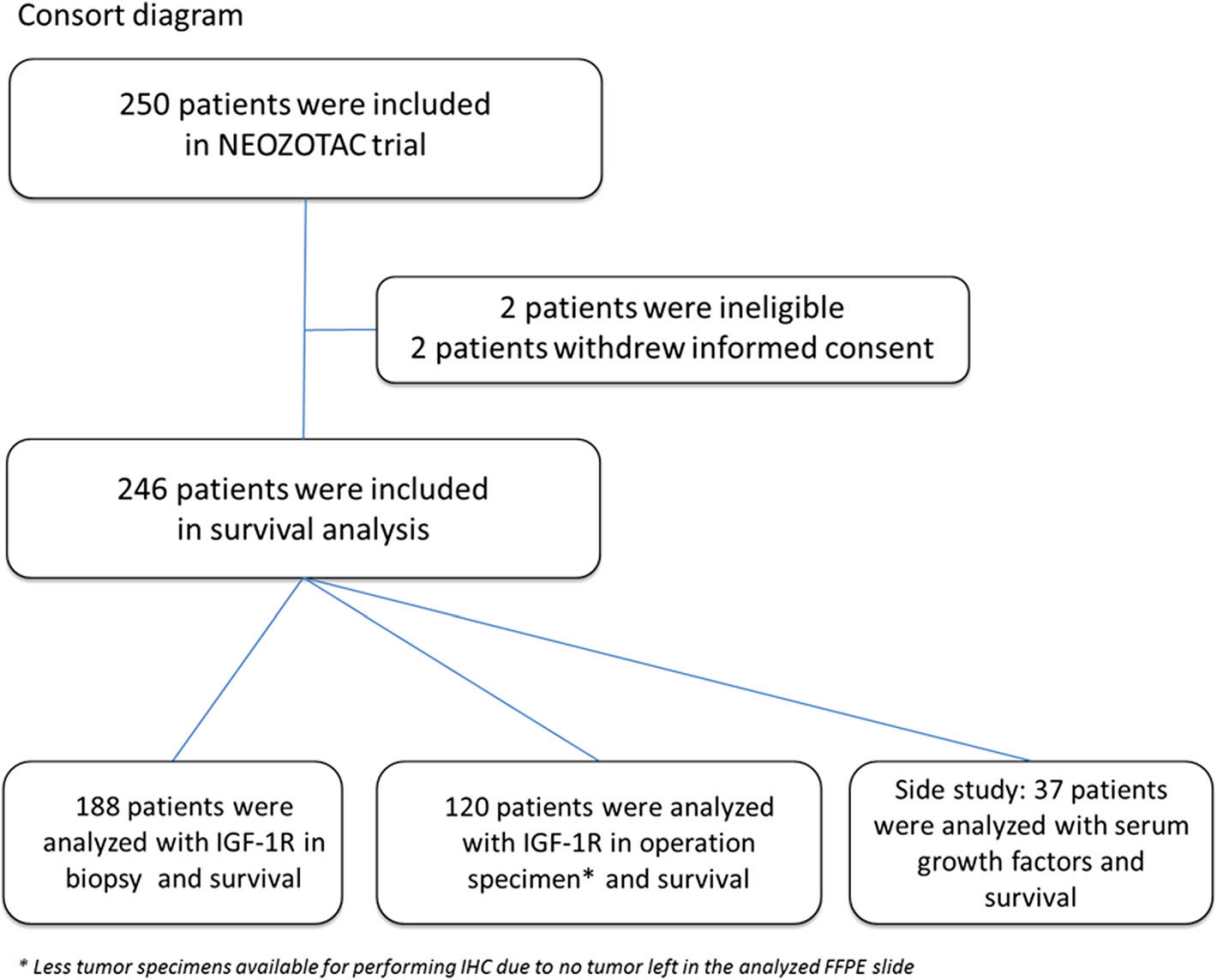
Consort diagram of the trial

As previously described [23], from July 2010 until April 2012, 250 women participated in the multi-center phase III NEOZOTAC trial and 246 were evaluated in the study (2 patients were ineligible and 2 patients withdrew informed consent; Fig. 1). Eligible patients had a histologically confirmed diagnosis of HER2 negative, stage II or III (T2 any cN, cM0) early breast cancer, adequate bone marrow (i.e., white blood cell count ≥ 3.0 × 10^9^/L, neutrophil count ≥ 1.5 × 10^9^/l, and platelet count ≥ 100 × 10^9^/l), normal liver function (i.e., bilirubin ≤ 1.5 × upper limit of normal (UNL) range, ALAT and/or ASAT ≤ 2.5 × UNL, alkaline phosphatase ≤ 5 × UNL), adequate renal function (i.e., calculated creatinine clearance ≥ 50 mL/min), adequate cardiac function, WHO performance state 0–2, age ≥ 18 years, absence of pregnancy or current lactation, and written informed consent. Menopause was defined as 1 year without menstrual activity, previous bilateral oophorectomy, age older than 60 years or baseline FSH > 20 U/l, and estradiol < 110 pmol/l. The study (NCT01099436) was conducted in accordance with the Declaration of Helsinki (October 2008) and was approved by the Ethics Committee of the LUMC in agreement with the Dutch law for medical research involving human subjects.

### Treatment

Women received 6 cycles of neo-adjuvant TAC chemotherapy (75 mg/m^2^ of docetaxel, 50 mg/m^2^ of doxorubicin and 500 mg/m^2^ of cyclophosphamide) with or without ZA (4 mg i.v. in 15 min within 24 h after chemotherapy, repeated every 21 days for 6 cycles). Pegfilgrastim (Neulasta®) was administered as primary prophylaxis (6 mg once per cycle) as a subcutaneous injection 24 h after chemotherapy for all cycles. ZA therapy was combined with daily supplements of 500 mg calcium and 400 IU vitamin D.

### Randomization

Patients were randomized at the LUMC Datacenter of the Department of Surgery using Pocock’s minimization technique, stratified by center, clinical T-classification, clinical N-classification, and estrogen receptor status. The ALEA randomization program was used.

### Immunohistochemistry

Formalin-fixed paraffin-embedded (FFPE) tumor tissue samples of pre-chemotherapy biopsies and surgical specimens were collected for analysis of IGF-1R expression using immunohistochemistry (IHC) (Fig. 1). The staining method is described extensively elsewhere [25].

### Blood sampling and analysis

Non-fasting blood samples were obtained directly before chemotherapy administration to measure glucose, insulin, and IGF-1 levels. Samples were collected and kept on ice directly after drawing. After centrifuging, the supernatant was stored at − 80 °C and, at the end of the study, was sent to the Leiden University Medical Center (LUMC) for analysis. Serum glucose levels were determined by spectrophotometry (Modular P800, Roche Diagnostics, Almere, The Netherlands), and insulin levels were analyzed with the chemiluminescence immunoassay (CIA) (Immulite 2500, Siemens, The Hague, The Netherlands). Serum levels of IGF-1 (IDS-iSYS) were analyzed with Immunodiagnostic Systems (Frankfurt, Germany). The IGF-1 assay is traceable to the WHO IS 02/254.17.

### Endpoints

The primary endpoint of the study was pCR, and the results of pCR are described elsewhere [23]. PCR was defined as the absence of residual invasive cancer within the breast and lymph nodes according to the Miller and Payne (MP) classification [28]. Secondary endpoints were DFS, defined as the time from date of inclusion until the date of the earliest documented local or distant recurrence, contralateral breast cancer including DCIS, second primary invasive cancer, or death from any cause, and OS, defined as the time from inclusion until date of death from any cause. Additionally, we studied the association between insulin, glucose, IGF-1 levels, and IGF-1R expression with survival outcomes.

### Statistical analysis

Median follow-up was calculated by applying the reverse Kaplan–Meier methodology. Cox regression models were used to evaluate the effect of ZA and other risk factors on DFS and OS. Hazard ratios (HRs) and 95% confidence intervals (95% CIs) were estimated. Relevant risk factors described in the literature or found to have a *P* value of less than 0.1 in univariate analyses were incorporated in the multivariate model. All tests were two-tailed. *P* values of less than 0.05 were considered significant. All analyses were computed using SPSS software™ 23.0 (IBM Corp., Armonk, NY, USA).

## Results

### Patient characteristics

The clinical characteristics of the patients included in the study are shown in Table 1 and were described previously [23]. None of these patient characteristics were significantly different between the two groups. Of the total cohort, 39.4% had a postmenopausal status at the start of the study.

**Table 1:** Patient characteristics

	TAC + ZA *N* = 122 (49.6%)	TAC *N* = 124 (50.4%)	IGF-1R biopsy data available *N* = 188 (76.4%)	Serum data available *N* = 37 (15%)
Median age (range), Years	48.0 (29–67)	49.0 (34–70)	49 (29–70)	49 (34–65)
Median BMI (range), kg/m^2^	26.1 (18.5–40.0)	25.0 (18.3–42.0)	25.0 (18.3–42.0)	24.9 (19.4–39.5)
Menopausal status				
Pre/peri	72 (59.0%)	75 (60.5%)	110 (58.5%)	24 (64.9%)
Post	50 (41.0%)	47 (37.9%)	76 (40.4%)	13 (35.1%)
T-classification				
T1/T2	73 (59.8%)	71 (57.3%)	108 (57.4%)	21 (56.8%)
T3/T4	49 (40.2%)	53 (42.7%)	80 (42.6%)	16 (43.2%)
N-classification				
N0	54 (44.3%)	56 (45.2%)	90 (47.9%)	22 (59.5%)
N+	68 (55.7%)	68 (54.8%)	98 (52.1%)	15 (40.5%)
HR-status				
ER+ and/or PR+	101 (82.8%)	104 (83.9%)	158 (84.0%)	33 (89.2%)
ER− and PR−	21 (17.2%)	20 (16.1%)	30 (16.0%)	4 (10.8%)

*TAC* docetaxel, doxorubicin and cyclophosphamide, *ZA* zoledronic acid, *BMI* body mass index, *HR* hormone receptor, *ER* estrogen receptor, *PR* progesterone receptor, *pCR* pathologic complete response, *LN* lymph nodes, *MP* Miller and Payne

### Response

**Fig. 2:**
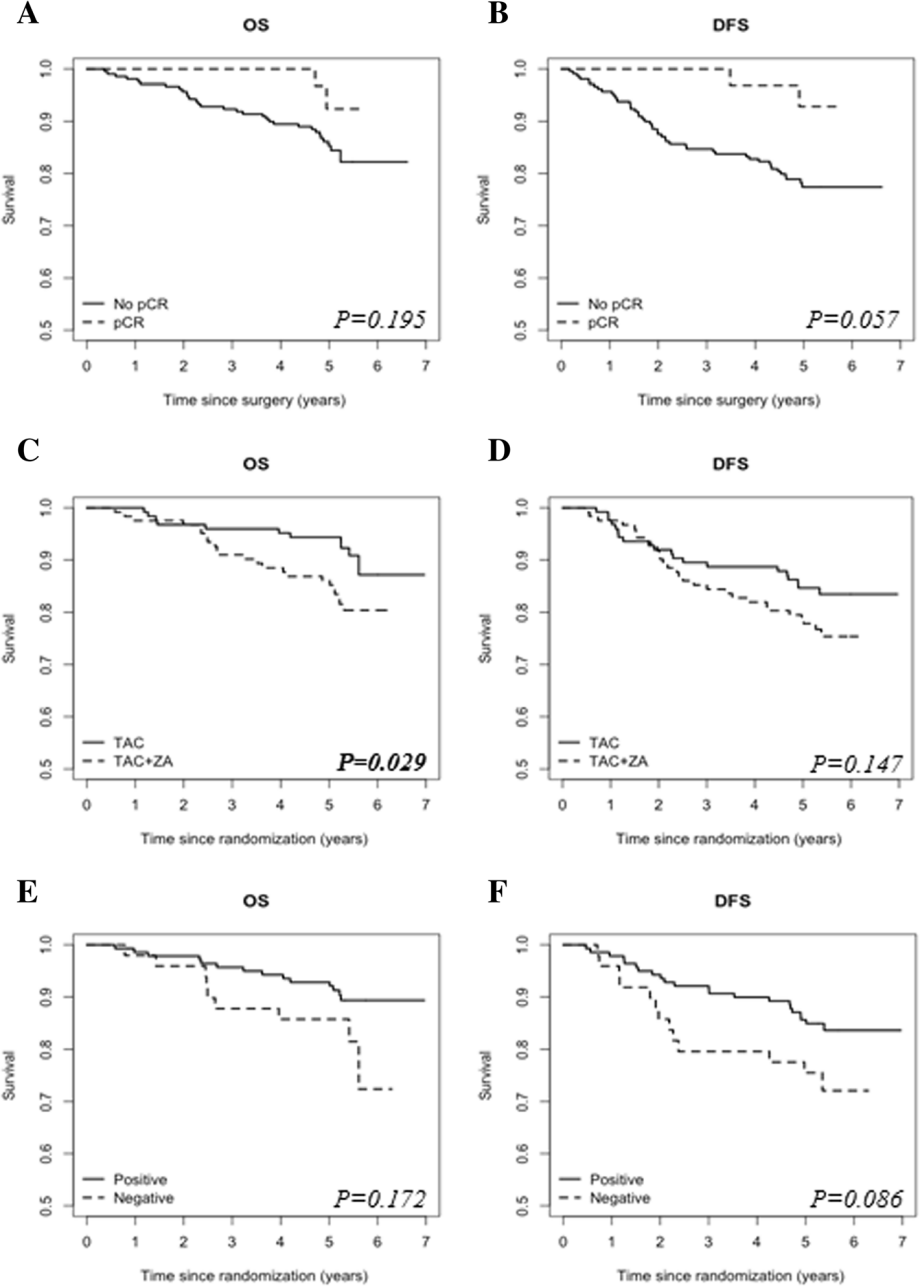
Kaplan–Meier curves of overall survival (left column) and disease free-survival (right column) for pCR (**a** and **b**), for treatment with or without zoledronic acid (**c** and **d**), and IGF-1R expression before neoadjuvant chemotherapy (**e** and **f**). Note: *P*-values are given for the univariate analyses of the Cox regression analyses. Bold values indicate that *P* < 0.05. Abbreviations: IGF-1, insulin-like growth factor 1; DFS, disease-free survival; OS, overall survival, pCR, pathological complete response

The primary endpoint pCR was achieved in 13.3% of the total cohort. This was not significantly different between the two arms (*P* = 0.980). As described previously, pCR was also not significantly different between the two arms in a subgroup analysis of postmenopausal women (14.0% versus 8.7%) [23]. The pCR and recurrence rates are shown in Table 2. Patients with pCR after neoadjuvant chemotherapy had a numerically longer period of DFS (HR 0.253, 95% CI 0.061–1.041, *P* = 0.057), but OS was not associated with pCR (HR 0.389, 95% CI 0.093–1.624, *P* = 0.195) (Fig. 2a, b).

**Table 2:** Short-term and long-term outcome

Response	TAC + ZA *N* = 122 (49.6%)	TAC *N* = 124 (50.4%)	*P* value
pCR breast and LN			
Yes	16 (13.3%)	16 (13.2%)	
No	104 (86.7%)	105 (86.8%)	*0.980*
Miller and Payne			
1	19 (15.8%)	18 (14.8%)	
2	35 (29.2%)	31 (25.4%)	
3	24 (20.0%)	25 (20.5%)	
4	21 (17.5%)	25 (20.5%)	
5	21 (17.5%)	23 (18.9%)	*0.950*
Recurrence			
Total	29 (23.8%)	20 (16.1%)	*0.134*
Local	5 (4.1%)	5 (4.0%)	*0.979*
Regional	7 (5.7%)	4 (3.2%)	*0.341*
Distant	27 (22.1%)	17 (13.7%)	*0.085*
Second primary tumor	5 (4.1%)	5 (4.0%)	*0.979*
Death			
Yes	23 (18.9%)	11 (8.9%)	
No	99 (81.1%)	113 (91.1%)	**0.023**
Cause of death			
Breast cancer	22 (95.7%)	11 (91.6%)	*0.630*
Other	1 (4.3%)	1 (8.3%)	

*TAC* docetaxel, doxorubicin, and cyclophosphamide, *ZA* zoledronic acid, *pCR* pathologic complete response, *LN* lymph nodes, *MP* Miller and Payne

the italicized data have a significance of > 0.05

### Survival outcomes

**Fig. 3:**
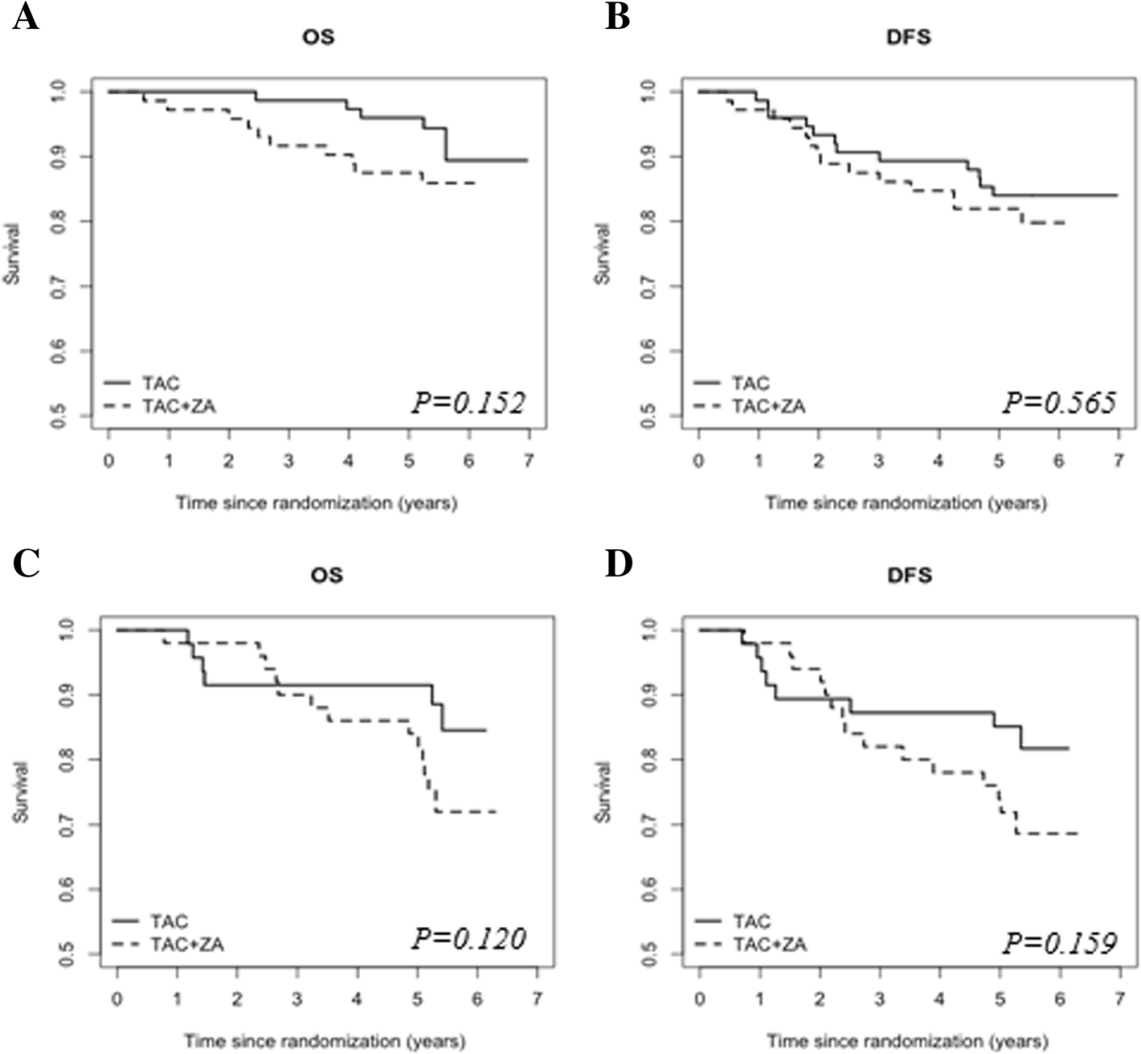
Kaplan–Meier curves of overall survival (left column) and disease-free survival (right column) for pre/perimenopausal women (**a**, **b**) and postmenopausal women (**c**, **d**). *P* values are given for the univariate analyses of the Cox regression analyses. DFS, disease-free survival; OS, overall survival

The median follow-up was 6.43 years (95% CI 6.25–6.61). Kaplan–Meier curves of survival rates are shown in Fig. 2. Risk factors associated with survival as described in the literature and those with *P* < 0.1 in univariate analyses were included in the regression model for multivariate analysis of mortality determinants. A Cox model was used to study the associations between risk factors and survival outcomes. The estimated HRs and associated 95% confidence intervals for univariate and multivariate analyses for OS and DFS are shown in Tables 3 and 4, respectively. Age, hormone receptor status, T status, N status, and menopausal status were adjusted for in the multivariate Cox model. Women who received ZA had a significantly worse OS than women who did not receive ZA in univariate analyses (HR 0.448, 95% CI 0.218–0.919, *P* = 0.029) (Fig. 2c) and in multivariate analyses (HR 0.468, 95% CI 0.226–0.967, *P* = 0.040). DFS did not significantly differ between groups in univariate analysis (HR 0.656, 95% CI 0.371–1.160, *P* = 0.147) (Fig. 2d). In the ZA arm, one patient died of stage IV lung cancer, and in the control arm, one patient died of unknown causes. The percentage of breast cancer deaths was not significantly different between both arms. In a subgroup analysis of postmenopausal women, the addition of ZA to chemotherapy did not affect DFS or OS (HR 0.539, 95% CI 0.228–1.273, *P* = 0.159; HR 0.464, 95% CI 0.176–1.222, *P* = 0.120, respectively) (Fig. 3a, b). There was also no significant difference in survival (DFS or OS) between the two arms in the premenopausal subgroup (HR 0.798, 95% CI 0.369–1.725, *P* = 0.565; HR 0.456, 95% CI 0.156–1.336, *P* = 0.152, respectively) (Fig. 3c, d).

**Table 3:** Univariate and multivariate Cox models of OS

	Univariate analysis			Multivariate analysis		
	HR	95% CI	*P* value	HR	95% CI	*P* value
Age	1.042	0.999–1.086	**0.054**	1.019	0.962–1.079	*0.522*
BMI	1.013	0.943–1.088	*0.730*			
HR status	2.019	0.942–4.328	**0.071**	*2.104*	0.978–4.529	*0.057*
Menopausal status	2.133	1.081–4.210	**0.029**	1.768	0.670–4.665	*0.250*
cN status	3.921	1.624–9.471	**0.002**	4.060	1.672–9.859	**0.002**
cT status	1.680	0.857–3.295	*0.131*	1.1516	0.763–3.011	*0.235*
Zoledronic acid	0.448	0.218–0.919	**0.029**	0.468	0.226–0.967	**0.040**

Bold values indicate that *P* < 0.05*OS* overall survival, *HR* hazard ratio, *CI* confidence interval, *BMI* body mass index

the italicized data have a significance of > 0.05

**Table 4:** Univariate and multivariate Cox models of DFS

	Univariate analysis			Multivariate analysis		
	HR	95% CI	*P* value	HR	95% CI	*P* value
Age	1.034	0.998–1.070	**0.061**	1.036	0.850–2.637	**0.043**
BMI	0.989	0.930–1.053	*0.739*			
HR status	1.698	0.868–3.323	*0.122*	1.799	0.916–3.536	*0.088*
Menopausal status	1.393	0.795–2.442	*0.247*			
cN status	2.724	1.420–5.224	**0.003**	2.811	1.461–5.407	**0.002**
cT status	1.569	0.896–2.748	*0.115*	1.497	0.850–2.637	*0.162*
Zoledronic acid	0.656	0.371–1.160	*0.147*			

Bold values indicate that *P* < 0.05

*DFS* disease-free survival, *HR* hazard ratio, *CI* confidence interval, *BMI* body mass index

the italicized data have a significance of > 0.05

### IGF pathway analysis

IGF-1R expression data was available for 188 patients before and 120 patients after chemotherapy treatment. The clinical characteristics of the patients included in the IGF pathway analysis are shown in Table 1 and were described previously [29]. The presence of IGF-1R expression in the tumor pre-treatment was numerically related to DFS, but this was not significant (HR 0.549, 95% CI 0.276–1.089, *P = 0.086*) (Fig. 2e), and it was not associated with OS (HR 0.562, 95% CI 0.246–1.285, *P = 0.172*) (Fig. 2f). In patients with HR-positive breast cancer, the presence of baseline IGF-1R tumor expression was associated with a better DFS in univariate analyses (HR 0.433, 95% CI 0.198–0.946 *P = 0.036*), but not in multivariate analysis (HR 0.484, 95% CI 0.214–1.096, *P = 0.082*). There was no significant association between the IGF-1R receptor and OS (HR 0.433, 95% CI 0.198–0.946 *P = 0.120*). Neither the presence of IGF-1R expression after neoadjuvant chemotherapy nor the decrease in expression during therapy was related to survival. Furthermore, treatment with ZA had no influence on the IGF-1R expression in the surgical specimen after chemotherapy treatment.

In a subgroup of patients (*N* = 37), baseline serum levels of glucose, insulin, and IGF-1 were measured. Patient characteristics are reported in Table 1. These were not significantly different compared to the total cohort. Lower serum insulin levels were associated with improved DFS (HR 1.025, 95% CI 1.005–1.045, *P = 0.014*), but not OS (HR 1.073 95% CI 0.953–1.209, *P = 0.244*). Glucose and IGF-1 concentrations were not associated with survival.

## Discussion

This study found that ZA as an adjunct to neoadjuvant chemotherapy had no beneficial effect in patients with stage II/III HER2-negative breast cancer receiving TAC chemotherapy and, in pre- and postmenopausal patients, was associated with worse OS, but not DFS. Additionally, in a post hoc analysis, there was no beneficial effect of ZA in postmenopausal patients. Interestingly, lower insulin levels were associated with improved DFS, but not with OS.

The negative impact of ZA on OS when used as an adjunct to neoadjuvant chemotherapy was not expected, as several studies have shown a benefit of ZA in the adjuvant setting in postmenopausal women [4]. Our study population may explain the negative impact of ZA on survival, as the majority (59.8%) of patients were premenopausal. Accordingly, the Azure trial showed that ZA in the adjuvant setting was associated with worse DFS and OS in a subgroup of patients younger than 40 years old, who are presumably largely premenopausal. [30]. However, we also did not find a benefit in postmenopausal patients.

Moreover, a major difference between adjuvant and neo-adjuvant use of BPs is the length of administration. Neoadjuvant BPs are administered for a shorter time period and therefore may not positively impact survival outcomes. In the JONIE1 trial, ZA did not have a beneficial effect on survival in the neoadjuvant setting [31], although the authors did find a positive association with pCR in previous studies [32]. In keeping with our results, a meta-analysis of four studies did not show any effect of ZA addition to neoadjuvant chemotherapy on pCR rate [16].

In a predefined additional exploratory side study, lower serum insulin levels were associated with improved DFS. In keeping with this result, patients with insulin levels greater than 13 μIU/mL had a twofold increased risk for disease progression compared to patients with insulin levels below this cut-off [33]. Goodwin et al. found that higher fasting insulin levels at baseline in breast cancer patients without diabetes were also associated with worse OS [34]. Higher insulin levels may give the tumor a growth advantage, as most breast tumors express the IGF-1R and IR-A, both of which are involved in proliferation and tumorigenesis and are associated with tumor progression [27, 35].

Our study has some limitations. We are aware that the sample size is small and the results should therefore be interpreted with caution. The majority of the patients included in this study were premenopausal women, but the positive effect of ZA on survival would be expected in postmenopausal women. Our post hoc analyses of postmenopausal women are not statistically powered, making it impossible to draw firm conclusions. Patients using BPs at baseline were excluded; however, the use of adjuvant BPs might have influenced the survival outcome, but this information is not available in our study. The sample size of the additional exploratory study of growth factors was small. However, the results of our study provide further evidence of the importance of the insulin and IGF-1R pathway in breast cancer.

## Conclusion

Our study does not support the use of ZA as an adjunct to neoadjuvant chemotherapy in patients with breast cancer.

## Data Availability

Not applicable.
